# Does nutritional status affect Parkinson's Disease features and quality of life?

**DOI:** 10.1371/journal.pone.0205100

**Published:** 2018-10-02

**Authors:** Nedim Ongun

**Affiliations:** Department of Neurology, Burdur State Hospital, Burdur, Turkey; Philadelphia VA Medical Center, UNITED STATES

## Abstract

**Objectives:**

The aim of this study was to determine the relationship between nutritional status and Parkinson's Disease (PD) features in association with depression, anxiety and quality of life in people with PD.

**Materials and methods:**

This study was conducted on 96 patients with idiopathic PD to whom the following scales were applied: Unified Parkinson’s Disease Rating Scale (UPDRS), 39-item PD questionnaire (PDQ-39), Hospital Anxiety and Depression Score (HADS), Mini Nutritional Assessment (MNA). The scales and measurements were applied to patients at their first assessment. Patients with malnutrition or at risk of malnutrition were assessed by the dietitian and nutrition nurse. These patients received nutritional support through personalized diet recommendations and appropriate enteral nutritional products, considering factors such as age, comorbidity, socioeconomic and cultural conditions. At the end of 6 weeks, the scales and measurements applied during the first visit were again applied to the patients.

**Results:**

A significant and inverse correlation was determined between mental (Spearman r:-0.510, p<0.001), activities of daily living (Spearman r:-0.520, p<0.001), motor (Spearman r:-0.480, p<0.001), complications (Spearman r:-0.346, p<0.001) UPDRS subdivisions and total scores (Spearman r:-0.644, p<0.001) and total MNA score. A significant and inverse correlation was found between all PDQ-39 subdomains and total MNA score (p<0.05). The highest inverse correlations were found in mobility (Spearman r:-0.690, p<0.001) and stigma (Spearman r:-0.570, p<0.001). Both depression (Spearman r:-0.631, p<0.001) and anxiety (Spearman r:-0.333, p<0.001) scores were determined to be inversely correlated with total MNA score. At the 6-week control visit, significantly lower scores were found in all subdivisions and in the total UPDRS score, PDQ-39 score and in the patients' anxiety and depression scores (p<0.05). MNA scores were found to be significantly higher in the assessment performed after 6 weeks of support for patients who had abnormal nutritional status at inception (p<0.001).

**Conclusion:**

PD motor and nonmotor functions, disease duration and severity are related to nutritional status. Quality of life was also shown to be affected by changes in the nutritional status. These results show that nutritional status assessment should be a standard approach in the PD treatment and follow-up processes.

## Introduction

Idiopathic Parkinson's Disease (PD) affects several aspects of patients' daily life because of its chronic nature [1]. Quality of life (QoL) is associated with quality of health and is recognized as an important treatment outcome of many conditions [2]. Therefore, health-related QoL has been considered an important outcome indicator for the management, care and progression of PD [3]. Many studies have investigated the impact of several variables on health related QoL in patients with PD, including disease severity, motor symptoms, nonmotor symptoms and demographic and socioeconomic characteristics [4–8]. Few studies, however, have included the effect of nutritional status on QoL features in patients with PD [9,10]. For example, poor nutritional status has been shown to result in a lower QoL in elderly individuals [11,12]; and given people with PD are at risk of malnutrition, malnutrition in PD might contribute to poorer QoL [10,13]. Depression is also highly prevalent among patients with PD [14,15] reducing the QoL of the affected individuals [16]. The aim of this study was to determine the relationship between nutritional status and PD features in association with depression, anxiety and QoL in people with PD.

## Materials and methods

This study was conducted on 96 patients with idiopathic PD which were recruited from an outpatient referral movement disorder clinic. This study was approved by Pamukkale University Medical School Non-Interventional Clinical Trials Ethics Committee. Investigation has been conducted according to the principles expressed in the Declaration of Helsinki. All of the collected data were stored according to the ethical guidelines of medical research. All patients were informed about the aims and procedures and provided their written informed consent to participate in this study. Participation in this study was voluntary and the patients were free to withdraw from the project whenever they wanted.

Patients assessed by the same neurologist who had specialized in movement disorders and fulfilled the United Kingdom Brain Bank criteria for the diagnosis of PD [17]. All of the PD patients who were eligible for this study were required to be 55 years or older. Patients with moderate to severe dementia (mini-mental state examination (MMSE<24) [18] were excluded from the study, as were those who were following special diets (calorie restriction, low protein, supplements such as vitamins B1/B6/B12/D/E, omega-3 etc.). Also, any patient with atypical Parkinsonism, diabetes, gastroenterological (surgical procedure, malabsorbtion, inflammatory bowel disease) and/or renal (nephritis, kidney failure) medical history, defined psychiatric medical history before PD were not eligible. All patients were screened for dysphagia and if tube feeding was necessary, the patient was excluded from the study.

Data was collected through face-to-face interviews with the patients along with clinical examinations by means of a checklist and questionnaires. The demographic variables (age and sex), co-morbidities and duration of PD were saved. Data were collected based on both participants’ self reports and their medical records. Clinical characteristics of PD were assessed using the Unified Parkinson’s Disease Rating Scale (UPDRS) [19]. The Hospital Anxiety and Depression Scale (HADS) questionnaire [20] was used to evaluate the characteristics of anxiety and depression. The health-related QoL was also assessed by means of the Parkinson’s disease quality of life questionnaire (PDQ-39) [21]. The mini nutritional assessment (MNA) questionnaire was applied together with anthropometric measurements to evaluate the nutritional status, All of the assessments were done when the patients were in the ‘On’ state. The scales and measurements were applied to the patients after they underwent detailed physical examination and routine examinations in the first assessment.

Nutritional support protocol is part of routine clinical care in our clinic for the patients with abnormal nutrition status. Patients with malnutrition or under the risk of malnutrition (patients with the MNA score below 23.5) were assessed by the dietitian and nutrition nurse. Patients and caregivers were educated by nutrition nurse to adjust and modify food and nutrition habits. All nutritional interventions were made according to The European Society for Clinical Nutrition and Metabolism (ESPEN) guideline [22]. Patients were advised to consume a balanced diet, with special attention to adequate intake of dietary fiber, fluids, and macro and micronutrients. Individualized support was also provided. The general recommendations were eating with frequent and small portions with a variety of foods, drinking at least 2000 mL water per day and getting 30–35 g/day fiber for each day. Oral nutritional supplements were recommended for patients with malnutrition (MNA score < 17) and who do not cover their nutritional requirements with an enriched diet. Vanilla, banana or chocolate flavored, liquid form oral nutritional supplements containing omega-3 fatty acids, arginine, RNA nucleotides and calcium beta-hydroxy methylbutyrate along with essential macronutrients were administered to the patients. Nutritional supplements were recommended according to patients' basic daily calorie and protein intake and their daily needs. Energy requirements were calculated using 25–30 kcal/kg body weight or the Harris Benedict equation. Vitamin B12, vitamin D and folic acid supplements were provided if needed. Patients with vitamin B12 level below 200 pg/mL were treated with intramuscular cyanocobalamin for five days. 50000 IU of vitamin D3 was administered to individuals with a 25(OH)D level below 20 ng/ml. 5 mg/day per-oral folic acid was administered to individuals with a folic acid level below 4 ng/ml. Because iron use affects levodopa, iron supplements were allowed to be taken at least two hours after medication use when indicated. Since levodopa was also affected by the protein in the diet, all patients were allowed to take levodopa on an empty stomach one hour before the meal. In terms of drug-meal interaction, redistribution of protein intake throughout the day (low protein breakfast and lunch and consumption of a second course—with no quantitative restrictions in terms of protein—only at dinner) was recommended for patients. Daily protein intake was set to 0.8–1 mg/kg of body weight. Meals and supplements were arranged as five parts carbohydrate for one part protein.

Patients were visited at their homes weekly by the same nutrition nurse. Patients who failed to continue to the diet program or supplementary products as well as the patients who were clinically unstable in 6-week follow-up were excluded from the study. At the end of six weeks, the scales and measurements applied in the first visit were again applied to the patients who remained in the study. Excluded patients were dropped from the analysis and only the patients who finished the entire study were analysed in baseline and follow-up data.

### Scales

#### Unified Parkinson’s Disease Rating Scale (UPDRS)

UPDRS is the most commonly used scale in the clinical study of PD [23]. It is used to identify the severity of PD in different aspects including non-motor symptoms (part I), activities of daily living (ADL) (part II), motor examination (part III) and drug complications (part IV). The UPDRS is scored from a total of 147 points where higher scores define worsening disability [19].

#### 39-item PD questionnaire (PDQ-39)

The PDQ-39 is the most commonly used scale for assessing health-related quality of life in PD patients. It contains 39 items assessing eight aspects of QoL in PD patients: mobility, ADL, emotional well-being, stigma, social support, cognitions, communication and bodily discomfort. All questions on the PDQ-39 are coded in a Likert-scale from 0–4 (0: never, 1: occasionally, 2: sometimes, 3: often, 4: always). Each individual's total score is calculated as follows: 100 x (the sum of the patient's scores in the 39 questions / 4 x 39). The domain scores are calculated in the same way as the total score. The total score for the PDQ-39 varies from 0 to 100 and higher scores define worsening condition. In this study, we used the Turkish-translated version of the PDQ39 questionnaire, which has been previously shown to have a high reliability [24].

#### Hospital Anxiety and Depression Score (HADS)

The HADS questionaire is used to evaluate the levels of anxiety and depression. It is comprised of 14 questions divided into two sections; seven questions are related to anxiety and the other seven focus on depression. Each questionnaire is worth 0–21 points, providing separate scores for either the anxiety or depression subscales. Responses are determined by adding up the sum of 0–3 scores for each question (0: not at all, 3: very often) [20].

#### Mini Nutritional Assessment (MNA)

The MNA is a rapid nutrition-assessment tool in order to identify the risk of malnutrition. It can be used as a combined screening and assessment tool [25]. The MNA questionnaire includes 18 items grouped into two sub-sections: six questions in the first section and twelve questions in the second section. The MNA scale includes body mass index (BMI), weight loss, arm and calf circumference, appetite, medication, general and cognitive health, dietary matters, autonomy of feeding, and self-perception of health and nutrition. The maximum score in the MNA questionnaire is 30. A total score of less than 17 points indicates ‘‘malnutrition”; scores of 17–23.5 indicate ‘‘at risk for malnutrition”; scores equal to or more than 24 points indicate ‘‘normal nutritional status” [26]. In this study, we have used the full format of the Turkish-translated MNA scale [27] and the two subgroups of patients with 'malnutrition' and 'at risk for malnutrition' were merged together in one group called 'abnormal nutritional status'.

### Statistical analysis

SPSS software version 21 (IBM, Chicago, IL, USA) was used to evaluate the data obtained from the scales. Numerical variables were indicated as mean and standard deviation (SD). The Kolmogorov–Smirnov test was applied to determine the normality of the distribution of the total MNA score and it was found to be non-normal. The univariate relationship between the total MNA score and other scales were assessed with Spearman's correlation test. For the scores obtained in the sub-group analysis, independent samples T test was used in the comparison of normal versus abnormal nutritional status. Multiple logistic regression analysis was conducted to determine the factors independently related to the nutritional status. Paired samples T test was used to compare the scores at the baseline and at the end of six weeks. A two tailed P value less than 0.05 was considered statistically significant. Significance thresholds for statistical tests were corrected for multiple comparisons. The confidence intervals and the exact P-values for each pre-specified outcome and also Bayes factor for the primary outcome were calculated.

## Results

112 patients were screened for the study at the beginning and 16 patients were excluded during the follow up. 11 patients were failed to continue to diet program and/or oral supplements and 5 patients were clinically unstable during the follow up. 96 patients with PD (40 women (41.7%), 56 men (58.3%)) were included in the study. The average age was found to be 63.68 ± 6.41 years at the time of inclusion to the study and 52.2 ± 6.88 years at the beginning of the disease. Average disease duration was determined to be 9.04 ± 3.62 years. The average total UPDRS score was 43.25 ± 13.86. All demographic and baseline clinical characteristics were presented in Table 1.

**Table 1:** Demographic and baseline clinical characteristics of the patients.

Variables	Value (n:96)
**Age-year (mean ± SD)**	
At study onset	63,68 ± 6,41
At disease onset	52,2 ± 6,88
**Gender (%)**	
Female	40 (41,7)
Male	56 (58,3)
**Level of Education (%)**	
Literate	18 (18,7)
Primary / Secondary	40 (41,7)
High School	22 (22,9)
University	16 (16,7)
**Duration of Disease-year (mean ± SD)**	9,04 ± 3,62
**Co-morbidities (%)**	
Diabetes	12 (12,5)
Hypertension	16 (16,7)
Cardiovascular Diseases	13 (13,5)
Osteoarthritis	14 (14,6)
**Anthropometric Measurements (mean ± SD)**	
Weight (kg)	72,7 ± 13,5
Height (cm)	173,2 ± 9,5
Body Mass Index (BMI) (kg/m^2^)	23,2 ± 4,2
Mid-arm Circumference (cm)	26,1 ± 4,1
Calf Circumference (cm)	32,0 ± 3,9
**UPDRS Score (mean ± SD)**	
Part I—mental	4,38 ± 2,19
Part II—ADL	15,94 ± 4,68
Part III—motor	18,29 ± 7,04
Part IV—complications	5,35 ± 1,66
Total	43,25 ± 13,86

SD: Standard Deviation, UPDRS: Unified Parkinson’s Disease Rating Scale, ADL: Activities of daily living

Baseline MNA, PDQ-39 and HADS scoring result values were presented in Table 2. The average MNA score was 21.59 ± 3.99. While 41 patients were under malnutrition risk, 24 patients were found to be malnourished. The highest scores of PDQ-39 sub-domains (the worst condition) was observed respectively in the stigma (28.33 ± 4.44), bodily discomfort (26.69 ± 4.98), mobility (24.75 ± 4.01) and ADL (24.56 ± 4.49) domains. In HADS scores, anxiety subdomain score was found to be 12.47 ± 3.49, and depression subdomain score was found to be 12.07 ± 3.21.

**Table 2:** Baseline scores of the scales (Nutritional status (MNA), disease-related quality of life (PDQ-39), anxiety and depression (HADS)) of patients (n:96).

Scale / Domain	Mean ± SD
**MNA (Total)**	21,59 ± 3,99
**PDQ-39-domains**	
Mobility	24,75 ± 4,01
Activities of Daily Living	24,56 ± 4,49
Emotional Well-being	22,88 ± 2,57
Stigma	28,33 ± 4,44
Social Support	17,14 ± 3,76
Cognition	22,89 ± 5,14
Communication	20,66 ± 2,92
Bodily Discomfort	26,69 ± 4,98
**HADS-domains**	
Anxiety	12,47 ± 3,49
Depression	12,07 ± 3,21

SD: Standard Deviation, HADS: Hospital Anxiety and Depression Score, PDQ-39: 39-item PD questionnaire, MNA: Mini Nutritional Assessment.

The relationship between baseline nutritional status and UPDRS, PDQ-39 and HADS scores was presented in Table 3. In UPDRS scoring, a significant and inverse correlation was determined between mental (Spearman r:-0.510, p<0.001), ADL (Spearman r:-0.520, p<0.001), motor (Spearman r:-0.480, p<0.001), complications (Spearman r:-0.346, p<0.001) subdivisions and total UPDRS (Spearman r:-0.644, p<0.001) and total MNA score. A significant and inverse correlation was determined between all PDQ-39 subdomains and total MNA score (p<0.05). The highest inverse correlations were determined respectively in the groups of mobility (Spearman r:-0.690, p<0.001), stigma (Spearman r:-0.570, p<0.001) and bodily discomfort (Spearman r:-0.510, p<0.001). Both depression (Spearman r:-0.631, p<0.001) and anxiety (Spearman r:-0.333, p<0.001) scores were determined to be inversely correlated with total MNA score while it is stronger in HADS depression subdomain.

**Table 3:** Correlations between the baseline scores of UPDRS, PDQ-39, HADS and total MNA score (n:96).

Scale	Domains	Correlation Index	MNA score
**UPDRS**	Part I—mental	Spearman r	-0.510*
		p-value	<0.001
	Part II—ADL	Spearman r	-0.520*
		p-value	<0.001
	Part III—motor	Spearman r	-0.480*
		p-value	<0.001
	Part IV—complications	Spearman r	-0.346*
		p-value	<0.001
	Total	Spearman r	-0.644*
		p-value	<0.001
**PDQ-39**	Mobility	Spearman r	-0.690*
		p-value	<0.001
	Activities of Daily Living	Spearman r	-0.490*
		p-value	<0.001
	Emotional Well-being	Spearman r	-0.450*
		p-value	<0.001
	Stigma	Spearman r	-0.570*
		p-value	<0.001
	Social Support	Spearman r	-0.380*
		p-value	<0.001
	Cognition	Spearman r	-0.470*
		p-value	<0.001
	Communication	Spearman r	-0.390*
		p-value	<0.001
	Bodily Discomfort	Spearman r	-0.510*
		p-value	<0.001
**HADS**	Anxiety	Spearman r	-0.333*
		p-value	<0.001
	Depression	Spearman r	-0.631*
		p-value	<0.001

*Correlation is significant at the 0.01 level (2-tailed).

UPDRS: Unified Parkinson’s Disease Rating Scale, HADS: Hospital Anxiety and Depression Score, PDQ-39: 39-item PD questionnaire, MNA: Mini Nutritional Assessment, ADL: Activities of daily living.

Patients with malnutrition or under malnutrition risk were grouped with ‘abnormal nutritional status’. The patients with normal nutritional status and abnormal nutritional status were compared in terms of clinical characteristics, UPDRS, PDQ-39 and HADS scores (Table 4). There was no significant age-related difference between the patients with abnormal nutritional status and the patients with normal nutritional status (p:0.988). While there was no significant difference in the female patients, male patients were determined to have abnormal nutritional status more frequently (p:0.007). Patients with abnormal nutritional status were found to have significantly lower education levels (p<0.001). Patients with abnormal nutritional status were found to have significant longer disease history than the patients with normal nutritional status (p:0.041). All subdivisions and total score of the UPDRS scale were understood to be significantly higher in the patients with abnormal nutritional status (p<0.001). In all sub-domains of the PDQ-39 scale, patients with abnormal nutritional status were was found to have significantly higher scores (all p<0.05). Also, patients with abnormal nutritional status were observed to have significantly higher anxiety and depression scores than the patients with normal nutritional status (p<0.001).

**Table 4:** Comparison of the baseline clinical characteristics, UPDRS, PDQ-39 and HADS scores regarding nutritional status (MNA).

Variable	Abnormal Nutritional Status (n:65) (mean ± SD)	Normal Nutritional Status (n:31) (mean ± SD)	*p*-value
**Age (year)**	64,21 ± 4,14	63,60 ± 5,77	0.988
**Gender—Female**	22 (%33,8)	18 (%58)	0.622
**Gender—Male**	43 (%66,2)	13 (%42)	**0.007**
**LOE-Literate, Primary, Secondary**	47 (%72,3)	11 (%35,4)	**<0.001**
**LOE-High School, University**	18 (%27,7)	20 (%64,6)	0.991
**Duration of Disease (year)**	11,12 ± 4,22	8,11 ± 3,40	**0.041**
**Body Mass Index (BMI) (kg/m**^**2**^**)**	22,1 ± 4,6	24,9 ± 4,1	**0.030**
**UPDRS—Part I—mental**	7,01 ± 3,08	3,20 ± 1,52	**<0.001**
**UPDRS—Part II—ADL**	18,22 ± 4,25	10,82 ± 3,14	**<0.001**
**UPDRS—Part III—motor**	20,46 ± 7,89	15,28 ± 5,39	**<0.001**
**UPDRS—Part IV—complications**	7,18 ± 2,82	3,55 ± 1,21	**<0.001**
**UPDRS—Total**	50,01 ± 14,82	39,41 ± 12,09	**<0.001**
**PDQ-39—Mobility**	32,49 ± 5,21	20,18 ± 4,03	**<0.001**
**PDQ-39—Activities of Daily Living**	34,80 ± 5,14	19,27 ± 3,57	**<0.001**
**PDQ-39—Emotional Well-being**	33,31 ± 3,87	18,01 ± 2,04	**<0.001**
**PDQ-39—Stigma**	40,14 ± 5,48	20,30 ± 3,87	**<0.001**
**PDQ-39—Social Support**	22,27 ± 3,87	14,57 ± 3,24	**0.020**
**PDQ-39—Cognition**	29,53 ± 5,91	18,07 ± 5,01	**<0.001**
**PDQ-39—Communication**	26,27 ± 3,28	17,47 ± 2,82	**0.043**
**PDQ-39—Bodily Discomfort**	32,08 ± 5,27	19,82 ± 4,27	**<0.001**
**HADS—Anxiety**	14,88 ± 3,82	9,91 ± 3,01	**<0.001**
**HADS—Depression**	14,09 ± 3,17	10,27 ± 3,24	**<0.001**

SD: Standard Deviation, LOE: Level of Education, UPDRS: Unified Parkinson’s Disease Rating Scale, HADS: Hospital Anxiety and Depression Score, PDQ-39: 39-item PD questionnaire, MNA: Mini Nutritional Assessment, ADL: Activities of daily living.

The multiple logistic regression analysis showed that the factors of depression (OR = 1.121, p = 0.011), UPDRS total score (OR = 0.685, p = 0.019) and male gender (OR = 1.287, p = 0.009) were independently related to malnutrition.

The patients with abnormal nutritional status in baseline assessment were compared in terms of UPDRS, PDQ-39, HADS and MNA scores at six weeks control after nutritional support (Table 5). It was found that significantly lower scores were obtained in all sub-divisions and total score of UPDRS scale (p<0.05). In all subdivisions of PDQ-39 scale, significantly lower scores were observed following the six-week nutritional support (p<0.001). Significantly lower scores were also obtained in the anxiety and depression scores of the patients (p<0.001). MNA scores were found to be significantly higher in the assessment performed after the six-week support for the patients who had abnormal nutritional status at the beginning (p<0.001).

**Table 5:** Comparison of the UPDRS, PDQ-39, HADS and MNA scores in baseline and six-week control of the patients with abnormal nutritional status (n:65).

Variable	Baseline (mean ± SD)	Six-week control (mean ± SD)	*p*-value
**UPDRS—Part I—mental**	7,01 ± 3,08	4,56 ± 1,28	**<0.001**
**UPDRS—Part II—ADL**	18,22 ± 4,25	15,32 ± 3,83	**0.034**
**UPDRS—Part III—motor**	20,46 ± 7,89	17,53 ± 5,01	**0.040**
**UPDRS—Part IV—complications**	7,18 ± 2,82	3,70 ± 1,11	**<0.001**
**UPDRS—Total**	50,01 ± 14,82	40,98 ± 11,27	**<0.001**
**PDQ-39—Mobility**	32,49 ± 5,21	22,11 ± 4,21	**<0.001**
**PDQ-39—Activities of Daily Living**	34,80 ± 5,14	20,67 ± 4,27	**<0.001**
**PDQ-39—Emotional Well-being**	33,31 ± 3,87	18,19 ± 2,84	**<0.001**
**PDQ-39—Stigma**	40,14 ± 5,48	19,30 ± 3,17	**<0.001**
**PDQ-39—Social Support**	22,27 ± 3,87	12,96 ± 2,74	**<0.001**
**PDQ-39—Cognition**	29,53 ± 5,91	20,17 ± 4,21	**<0.001**
**PDQ-39—Communication**	26,27 ± 3,28	17,81 ± 2,81	**<0.001**
**PDQ-39—Bodily Discomfort**	32,08 ± 5,27	15,74 ± 3,72	**<0.001**
**HADS—Anxiety**	14,88 ± 3,82	9,07 ± 3,73	**<0.001**
**HADS—Depression**	14,09 ± 3,17	9,20 ± 3,16	**<0.001**
**Body Mass Index (kg/m**^**2**^**)**	22,1 ± 4,6	24,0 ± 3,8	**0.031**
**Mid-arm Circumference**	23,2 ± 3,2	24,1 ± 3,6	0.641
**Calf Circumference**	30,8 ± 3,7	31,1 ± 3,3	0.703
**MNA—total**	16,85 ± 2,54	22,37 ± 3,88	**<0.001**

SD: Standard Deviation, UPDRS: Unified Parkinson’s Disease Rating Scale, HADS: Hospital Anxiety and Depression Score, PDQ-39: 39-item PD questionnaire, MNA: Mini Nutritional Assessment, ADL: Activities of daily living.

## Discussion

According to the data obtained from our study, 67.7% of our patients had an abnormal nutritional status. In all subgroups of UPDRS and PDQ-39 scale which are used to define the severity of the disease and health related QoL in PD, higher scores were determined to be related to a poorer nutritional status. Similarly, people with lower MNA scores were found to have higher anxiety and depression scores. Depression, UPDRS score and male gender were determined to be independently related to malnutrition. A significant improvement was achieved in disease severity, QoL and nutritional status following nutrition education and support provided for patients with an abnormal nutritional status.

In our study, 41 (42%) patients were at risk of malnutrition and 24 (25%) patients had malnutrition. These numbers were higher than many previous studies [28–32]. The valid and reliable MNA scoring used in our study had been used in many studies previously conducted with different age and disease groups [28,33–35]. The primary reason for the difference in our results for nutritional status is most likely the different population in our study. Based on our experience, we think that, our study population does not typically have a balanced and adequate diet in terms of macro- and micronutrients. Due to their low income levels, nutritional intake is mostly based on carbohydrate. Education level is also important in terms of the ability to associate health problems with malnutrition and to find solutions. The fact that the population assessed in our study generally consisted of low-income patients with lower education levels and residing in rural areas could account for the higher malnutrition rates.

Patients with malnutrition and those at risk of malnutrition were observed to have more severe symptoms in all subgroups of the UPDRS scale including mental, motor, ADL and complications. Higher UPDRS scores were obtained from malnourished participants in a study in which a different nutritional status scale (subjective global assessment) was used and in another study in which the MNA was used [9,36]. PD severity is assessed from many aspects by the UPDRS. Nonmotor features were found to be directly related to the nutritional status as assessed by other scales in our study. In the sections assessing motor features, higher scores were obtained probably because of malnutrition-related loss of muscle strength and muscle mass, cramps and loss of balance. Eating and digestion difficulties that emerged in the course of PD were also thought to influence this condition.

Higher PDQ-39 scores were obtained in patients with abnormal nutritional status as well as in the QoL assessment. In other words, among the patients with PD included in our study, those with poor nutritional status were determined to have a poorer QoL. The strongest relationship with nutritional status was observed in the mobility subdomain and the result was similar to that of previous studies [9,10]. PD can lead to a reduction in mobility which affects nutrition related daily activities such as shopping and preparing meals. Moreover, malnutrition also reduces mobility and functional status and considerably decreases QoL [11,37–40]. In our study, aspects of QoL found to have the second strongest relationship with nutritional status was stigma. It is possible that the stigmatization felt in the community due to retarded physical motions caused by PD lead to a decreased QoL, eating avoidance and malnutrition. As nutritional status improves, the stigma is also significantly and positively affected due to increased self-confidence and improvement of other QoL indicators. Another QoL indicator, bodily discomfort is also closely related to nutritional status. The pains, cramps and aches felt significantly more by patients with a poor nutritional status can be explained by the neuropathic pain caused by the disease itself and particularly by nutritional deficiencies. This idea is supported by the fact that these negative feelings are reduced in patients with improved nutritional status. New placebo controlled studies, especially those performed with biochemically measurable nutritional indicators, are needed in this area. Social support is another QoL indicator with an important role in nutritional status [41]. We think that, patients with poor social support may have a lower food intake and malnutrition risk can contribute depression. This result can be assessed together with the fact that depression is more frequently observed in malnourished patients [28].

In our study, patients with PD who had a poor nutritional status were determined to have greater depression and anxiety. The data obtained were similar to previous studies showing the relationship between psychiatric findings and nutritional status [13,30,42]. In patients with PD, depression and anxiety were found to be a significant predictor of nutritional status. In these studies, both depression and anxiety were found to be related to malnutrition risk in patients with PD, and higher depression and anxiety scores were found in malnourished patients with PD [27,43]. Depression was also found to be an independent malnutrition risk factor in our study. Depression and anxiety cause reduced food intake, and the resulting weight loss and psychiatric findings could lead to a vicious cycle. The decrease in enthusiasm for daily activities such as shopping and meal preparation in patients with PD due to retarded physical motions could also lead to reduced food intake. In our study, the factors of depression, UPDRS total score and male gender were determined to be independently related to lower MNA scores or higher nutritional deficiency. Unlike previous studies [10,44], male gender was found to be a risk factor for malnutrition in our study. This result might stem from differences in the social structure and the fact that the assessed patient group mostly resided in rural areas.

In our study, a significant increase in total MNA score was observed after six weeks of nutritional support. MNA is a validated screening tool that assesses nutritional status from many aspects. In our study, an increase in all MNA subscores was observed. An increase in the mid-arm and calf circumference measures, an indicator of muscle mass, was observed. Statistically significant improvements were found in the 'dietary', 'subjective' and 'functional' subgroups. A clinically and statistically significant improvement in self perception of nutritional and health status could also be associated with a decrease in depression and anxiety scores. In addition, being involved in a nutritional intervention can affect mood and outlook. Eating positively affects mood by releasing endorphins, alleviating the cognitive effects of hunger and dehydration. Therefore, nutritional interventions appear to directly affect mood and outlook however, further placebo controlled studies are needed to support this finding.

An inverse relationship was found between MNA score and disease duration in our study. Patients with long term PD had lower MNA scores. Although a previous study found no relationship between disease duration and nutritional status [28], some studies found a direct relationship between disease duration and nutritional status which is consistent with our study [10,29]. Significantly lower scores were obtained in the UPDRS subdivisions and the total UPDRS score in the assessment conducted after the 6-week nutritional intervention. Similarly, significant positive changes were observed in PDQ-39 scores and HADS scores in the 6-week period. The data obtained in our study were similar to those obtained after a 12-week follow-up in a previous study [9]. It is important for the obtained data to be generalizable that there was a much larger study group in our study.

Patients were cared for weekly in person, within the 6-week period in our study, and when needed, effective and efficient nutritional support was provided through phone calls. Regular visits by the care team may have increased patient compliance and treatment participation. In addition, participation in the study also resulted in significantly improved emotional well-being. This may be explained by the placebo effect. A previous study showed that patient participation and effective communication increased patient satisfaction and treatment benefits in medical treatment decisions [45]. Supporting that result, our study showed that effective and close nutritional follow-up positively affected both patient satisfaction and treatment benefit.

Our study has some limitations. First, nutritional status indicators such as blood-serum measurements, daily food intake and fat and muscle mass measurements to assess nutritional status were not used. However, as an innovative clinical assessment tool, MNA scoring enabled the assessment of patients from several angles, because of the range of questions it contains. Second, only outpatient PD patients, i.e. PD patients with mild to moderate stages were included in our study. Patients with end stage PD who could be at a greater risk of malnutrition could not be included. Additionally, patients with moderate to severe dementia which is a known risk factor for poor nutritional status were excluded. Therefore, it would be more appropriate to generalize the data from our study to patients with mild to moderate stage PD without severe dementia who applied to outpatient clinics. Third, there is no placebo group in our study. But the lack of a placebo group is also a limitation for most nutrition intervention studies because of the difficulties in study design and ethical restrictions. However, since there is no placebo control group, it is difficult to say that the improvement in nutritional status has a direct effect on Parkinson's Disease features and quality of life characteristics. Despite these limitations, our study is the first study to assess nutritional status among Turkish patients with PD in terms of several QoL indicators and psychiatric status, by using validated and reliable MNA scoring instead of the BMI and weight measurement methods used in previous studies. In addition, we had a large sample size.

In summary, the data obtained in our study showed that nutritional status influenced PD-related factors. PD motor and non-motor features, disease duration and severity are related to nutritional status. QoL was also shown to be affected by changes in nutritional status. These results show that nutritional status assessment should be a standard approach in PD follow-up and treatment process. Although further research is still needed on the role of nutrition in the progression of neurodegenerative diseases, disregarding the nutritional features in PD theoretically worsens the disease process and QoL. However, follow-up and biochemical measurements performed on patients especially at various severity stages of the disease can help extensively reveal the relationship between PD and nutritional status.
